# Does country of residence matter? A cross-sectional comparison of PTSD and depression among traumatized, treatment-seeking Syrians residing in Syria and Syrian refugees in Germany and Turkey

**DOI:** 10.1016/j.jmh.2026.100399

**Published:** 2026-01-28

**Authors:** Max Bringmann, Maya Böhm, Freya Specht, Max Vöhringer, Majdy Aldoibal, Christine Knaevelsrud, Birgit Wagner, Maria Böttche, Yuriy Nesterko

**Affiliations:** a Department for Traumatic Stress and Transcultural Studies, Center ÜBERLEBEN Berlin, Berlin, Germany; b Clinical Psychological Intervention, Freie Universität Berlin, Berlin, Germany; c Clinical Psychology and Psychotherapy, Medical School Berlin, Berlin, Germany; d Department for Medical Psychology and Medical Sociology, Medical Faculty, University of Leipzig, Leipzig, Germany

**Keywords:** Syria, Refugees, Mental health, Trauma, SWANA

## Abstract

**Background:**

Refugees, including Syrians, exhibit higher rates of posttraumatic mental disorders than non-refugee populations, partly due to traumatic events and stressors before, during, and after displacement. However, differences in symptom load cannot solely be attributed to being a refugee, as comparison groups vary in other characteristics, such as country of residence and origin. Using a cross-country comparative design, the present study examined mental health outcomes associated with being a refugee by contrasting Syrian refugees with Syrian residents.

**Methods:**

Syrians residing in Syria (SRS) were compared with Syrian refugees in Germany (RSG) and Turkey (RST) regarding posttraumatic stress disorder (PTSD; using the PTSD Checklist for DSM-5), depression (using the Patient Health Questionnaire-9 (PHQ-9)), and traumatic event exposure (using a curated list of traumatic events). Participants (*n* = 689 total, *n* = 236 SRS, *n* = 254 RSG, *n* = 199 RST) were recruited from an open-label dissemination study between 2021 and 2024. Prevalence rates and trauma exposure were compared between groups, and mediation models examined trauma exposure as a potential mechanism underlying group differences, adjusting for employment, education, family status, age, and gender.

**Results:**

Prevalence rates were calculated for depression (SRS: 90%, RSG: 91%, RST: 86%) and PTSD (SRS: 75%, RSG: 81%, RST: 72%), with no significant differences between refugees and Syrian residents. However, both refugee groups reported significantly more trauma load (SRS vs. RSG: *t* = -5.94, *p* < 0.001; SRS vs. RST: *t* = -4.87, *p* < 0.001). Mediation models indicated that trauma load partly explained existing differences in mental health outcomes between refugees and Syrian residents (Indirect effects - SRS vs. RSG for PTSD: *β* = 0.040, 95% CI [0.015, 0.065], *p* = 0.002; SRS vs. RST for depression: *β* = 0.028, 95% CI [0.003, 0.053], *p* < 0.03; SRS vs. RST for PTSD: *β* = 0.059, 95% CI [0.028, 0.090], *p* < 0.001).

**Conclusion:**

PTSD and depression rates did not differ between refugees and Syrian residents from Syria, emphasizing the high mental health symptom burden in treatment-seeking Syrians regardless of their country of residence. Notably, though trauma exposure was higher among refugees and explained existing differences in symptom load, underscoring the need for psychosocial support across host countries.

## 1 Introduction

The number of forcibly displaced people worldwide has increased continuously, with historic highs reported year on year by the United Nations High Commissioner for Refugees (UNHCR), reaching almost 120 million in 2023 (UNHCR. Global Trends Report 2022 2023). Across the different stages of flight, refugees are affected by a wide range of stressors and traumatic events, such as experiencing combat, persecution, harsh transit conditions, and, once arrived in the country of current residence, discrimination, economic hardships, limited access to job market and health system as well as uncertainty around residence status (Bruhn et al., 2018; Pfeiffer et al., 2022; Steel et al., 2017; Theisen-Womersley, 2021).

The load of traumatic events experienced pre-, peri‑, and post-flight is particularly high in refugees. In two studies investigating non-treatment-seeking refugees in Norway (Lie et al., 2001) and Switzerland (Heeren et al., 2012) using the same list of traumatic events from the Harvard Trauma Questionnaire (Mollica et al., 1992), participants reported a mean number of different traumatic event types of 5.2 and 5.7, respectively, with separation from family/friends, war, and self-experienced or observed physical violence among the most commonly mentioned traumatic experiences. Other studies assessing treatment-seeking refugee populations and/or using different questionnaires have found even higher averages of the exposure of traumatic events (Jongedijk et al., 2020; Weathers et al., 2013).

Given these psychological stressors, research has been conducted to understand the physical and mental illness burden of refugees and asylum-seekers in different host countries (Fazel et al., 2005; Hoell et al., 2021). Specifically, over the past decades, multiple studies have examined the prevalence of mental health conditions in refugees, with a particular emphasis on depression and post-traumatic stress disorder (PTSD). A meta-analysis reported prevalence rates of 4.0–6.0 % for depression and 8.0–10.0 % for PTSD in refugees (Fazel et al., 2005), while others found notably higher numbers of 25.0–45.0 % for depression and 31.0–63.0 % for PTSD (Lindert et al., 2009; Steel et al., 2009). In a more recent analysis, Henkelmann and colleagues (2020) found prevalence rates of 30.0 % and 40.0 % for diagnosed and self-reported depression, respectively, and prevalence rates of 29.0 % and 37.0 % for diagnosed and self-reported PTSD (Henkelmann et al., 2020). Altogether, prevalence rates for PTSD and depression among refugees and asylum-seekers seem to range between 30.0 % and 40.0 % in high-income Western countries.

While these results suggest a high exposure to traumatic events and symptom load in refugees, gauging the mental health impact of being a refugee requires an appropriate group of comparison. Comparing refugees to non-refugees living in Western countries or regions of origin (e.g. Southwest Asia and North Africa [SWANA]) has a caveat: the comparison groups differ either concerning the country of origin or residence as well as refugee status. Crucially, country of origin and country of residence – including cultural influences, socioeconomic environment, access to health services etc. – may impact symptom load for different mental disorders (Agic et al., 2019; Berry, 1997; Choy et al., 2021). For instance, Berry (1997) described different acculturation strategies migrants use regarding adopting/not adopting the host country’s culture while retaining/not retaining their own (Berry, 1997). A recent meta-analysis found that marginalisation – i.e., rejecting one’s own and the host country’s culture – was associated with higher depressive symptom severity compared to other acculturation strategies (Choy et al., 2021).

Considering between-country variability, there is a need to more clearly understand the impact of being a refugee on mental health by using a suitable group of comparison. One potential solution would be to compare individuals from the same country of origin currently living in different countries (i.e., residents of one particular country of origin as well as refugees of the same origin living in different host countries), thus eliminating variation from various countries of origin while accounting for differences in residence country.

Syrian people constitute one group that has been particularly affected by war, forced displacement and conflict for more than a decade and, as a result, has seen large parts of its population being internally displaced and fleeing the country (UNHCR. Global Trends Report 2022 2023). In a study on over 1000 Syrian refugees resettled in Sweden and sampled from a population registry (Tinghög et al., 2017), participants reported a mean of 4.0 different traumatic events pre-flight and 2.1 peri‑flight using the refugee trauma history checklist, with war, separation from and loss of family/friends as the most frequent event types. In a registry-based study with Syrian refugees in Germany (Georgiadou et al., 2018), a mean of 2.2 traumatic events was found using the Essen Trauma Inventory (Tagay et al., 2007), with military conflict and death of a loved one among the three most frequent event types next to serious accident/explosion. Other studies investigating treatment-seeking Syrian refugees or Syrian refugees resettled in non-Western countries found either similar or higher rates of trauma exposure (Alsamman et al., 2024; Renner et al., 2021). Overall, despite differences in sample composition, country of residence, and assessment methods, these studies indicate a considerable trauma load in Syrian refugees residing outside of Syria.

Beyond trauma load, studies have revealed a range of prevalence rates for common mental disorders in Syrian refugees. A recent meta-analysis including host countries in Europe (e.g., Germany, Sweden, Greece), the Southwest Asia (e.g., Jordan, Lebanon, Iraq), North, and South America (e.g., USA, Canada, Brazil) reported PTSD prevalence rates ranging from 26.0 % to 84.0 %, and a depression prevalence range from 34.7 % to 59.4 % (Sá FH de et al., 2022). Another study considering only Syrian refugees residing in Middle Eastern or European countries found slightly lower rates, with 43.0 % for PTSD and 40.9 % for depression (Peconga and Høgh Thøgersen, 2020). A separate study including only Syrian refugees in high-income Western European countries reported prevalence rates of 31.0 % for both PTSD and depression (Nguyen et al., 2022). Thus, PTSD and depression prevalence rates for Syrian refugees are very high, with rates for PTSD typically surpassing those for depression and a substantial between-country and between-study variation.

Turkey and Germany have been common host countries for Syrian refugees, ranking among the top host countries in recent years (UNHCR. Global Trends Report 2022 2023). Research results on mental health in Syrian refugees in these host countries has been similarly wide-ranging. In a register-based follow-up study on the prevalence of common mental disorders among Syrian refugees in Erlangen, Germany, 26.9 % were found to have clinically relevant depression, and 13.9 % had PTSD (Borho et al., 2020). In a more recent publication on Syrian refugees resettled in Leipzig, Germany, Schoenberger et al. (2024) reported a depression prevalence rate of 28.7 % and a PTSD prevalence rate of 25.3 % (Schoenberger et al., 2024).

Even more research has focussed on the mental health of Syrian refugees resettled in Turkey, providing similarly heterogeneous results. In a study among over 1500 Syrian refugees in Sultanbeyli-Istanbul, Turkey, 19.6 % of respondents screened positively for PTSD, and 34.7 % for depression (Fuhr et al., 2019). In another study (Kaya et al., 2019) conducted in Ankara, Turkey, prevalence rates were 36.5 % and 47.7 % for PTSD and depression, respectively. Additionally, in an online survey of Syrian refugees in Turkey, prevalence rates of 41.1 % and 41.6 % were reported for depression and PTSD, respectively (Kurt et al., 2021).

Even though not many studies on the mental health exist among Syrians who remained in Syria, they are pointing to elevated rates of PTSD and depression. For instance, Al Ibraheem and colleagues (2017) reported a prevalence of 31.8 % for PTSD in a sample of 195 internally displaced Syrians in Aleppo and Idlib (Al Ibraheem et al., 2017). Moreover, Tekeli-Yesil et al. (2018) found a depression prevalence rate of 70.5 % and a PTSD prevalence rate of 29.8 % among internally displaced persons in Syria (Tekeli-Yesil et al., 2018). Overall, studies on Syrians residing both in and outside of Syria show high prevalence rates for PTSD and depression, with considerable variability across studies.

Taken together, research on mental health among refugees has so far mainly investigated refugees from different countries of origin in various host countries. From these studies, the impact of being a refugee on mental health has been gauged by comparing refugees to non-refugee samples from mostly Western host countries or countries of origin in the SWANA region. In these comparisons, differences between refugees and general populations of common host countries are confounded with differences in country of origin and residence. Comparing a single refugee group (e.g., Syrians) across host countries may be a way of dealing with this confoundedness. To date, several studies on the mental health of Syrian refugees in different countries of residence have been conducted, including in Syria, Turkey, and Germany. However, to the best of our knowledge, none of these studies have compared these groups using cross-country research methods. The present study aims to fill this gap. Particularly, Syrians residing in Syria (i.e. Syrians residing in Syria; SRS) were compared to Syrian refugees in Germany (i.e. refugees from Syria in Germany; RSG) and Turkey (i.e. refugees from Syria in Turkey; RST). We hypothesised that Syrian refugees outside of Syria would display higher prevalence rates of PTSD and depression, given the potential additional traumatic events experienced during and after fleeing. To further investigate this hypothesis, we examined whether the number of traumatic events explained the association between being a Syrian refugee outside of Syria versus a Syrian resident and depression/PTSD symptom severity. Of note, Syrians residing in Syria may include individuals that have experienced displacement even if not across state borders. To account for this differentiation, Syrian refugees in Turkey or Germany are termed Syrian international refugees or Syrian refugees outside of Syria.

## 2 Methods

### 2.1 Procedure and participants

This cross-sectional study is part of a broader open-label dissemination treatment study evaluating the efficacy of internet-based psychotherapeutic interventions for Arabic-speaking adults with posttraumatic stress symptoms or symptoms of depression. A detailed description of the treatment programme can be found in Stein et al. (2023) and El-Haj-Mohamad et al. (2024) (Stein et al., 2023; El-Haj-Mohamad et al., 2024). It is accessible to Arabic-speaking individuals, irrespective of their place of residence or origin. Recruitment was conducted between February 2021 and July 2024 through various online channels, including social media advertisements, collaborations with local partner organizations, word-of-mouth referrals, and a programme website. The website provided general information about PTSD, depression, available treatment options, and the purpose of the study. Participants were informed that their participation was entirely voluntary and that their data would be protected by strict security measures.

To be included in the present secondary analysis, participants had to be able to read and write in Arabic, have internet access, be at least 18 years of age, indicate Syria as their country of origin, and Syria, Turkey, or Germany as their current country of residence. Thus, the study sample consisted of Arabic-speaking adults originating from Syria and seeking internet-based psychological treatment for posttraumatic stress disorder or depression. Applying these criteria resulted in an overall sample size of *n* = 789 (*n* = 236 SRS, *n* = 254 RSG, *n* = 199 RST). After providing written informed consent, participants completed an initial assessment that gathered sociodemographic data, details on trauma exposure, and psychological symptom measures such as depression and PTSD. This baseline data was used for the current study and as a baseline assessment for their participation in the intervention. The study was reviewed and approved by the ethics committee at Freie Universität Berlin (126/2016 and 185/2018).

### 2.2 Assessment

All measures were administered online through a secure web portal. For instruments that were not yet available in standard Arabic at the time of the study's planning, a structured translation process was used. Initially, a native speaker translated the measures, followed by a back-translation conducted by an independent individual unfamiliar with the original version. A team of professionals then reviewed and discussed any discrepancies between the two versions and agreed upon a final version of each assessment. When necessary, instructional texts were modified to align with the online format.

### 2.3 Sociodemographic characteristics

The study included the following sociodemographic characteristics: Gender (male/female), age, family status (in a relationship/not in a relationship), educational background (low education/high education), and employment status (employed/unemployed). Additionally, being a refugee was assessed using a dichotomous yes/no question asking if a person had to leave his/her country and/or was unable to return to it due to persecution because of race, religion, nationality, belonging to a social group or political conviction or if the person was fleeing from violent conflict.

### 2.4 Traumatic experiences

Exposure to traumatic events was assessed using a compiled list that incorporated items from the Harvard Trauma Questionnaire (HTQ) (Mollica et al., 1992), the Posttraumatic Diagnostic Scale (PDS) (Foa et al., 1997), and the Life Events Checklist for DSM-5 (LEC-5) (Weathers et al., 2013). The final list included 24 items covering various potentially traumatic experiences. Following DSM-5 guidelines, participants indicated exposure to a traumatic event if they had personally experienced it, witnessed it happening to someone else, learned about it occurring to a close family member or friend, or encountered it as part of their professional duties. To measure cumulative trauma exposure, the total number of traumatic event types was calculated, representing the variety of different traumatic experiences without accounting for their frequency.

### 2.5 Posttraumatic stress symptoms

Posttraumatic stress symptoms experienced in the past month were evaluated using the PTSD Checklist for DSM-5 (PCL-5) (Weathers et al., 2013), a 20-item self-report questionnaire that aligns with DSM-5 PTSD criteria. Each item was rated on a five-point scale (0 = “not at all” to 4 = “extremely”), with higher scores reflecting greater symptom severity. A total symptom severity score ranging from 0 to 80 was calculated, and scores above 32 indicate the presence of a probable clinical PTSD. The PCL-5 has been successfully validated as a screening tool for Arabic-speaking individuals (Ibrahim et al., 2018). In the present sample, the measure demonstrated high internal consistency with a Cronbach’s alpha of 0.90.

### 2.6 Depressive symptoms

Depressive symptoms over the past two weeks were measured using the Arabic version of the Patient Health Questionnaire-9 (PHQ-9) (Kroenke et al., 2001; Sawaya et al., 2016; Spitzer et al., 1999). This nine-item self-report instrument assesses core symptoms of depression, with responses rated on a four-point scale from 0 ("not at all") to 3 ("nearly every day"). A total score was computed, with higher values indicating more severe depressive symptoms and scores above 9 indicating moderate depression. The PHQ-9 has been shown to have good internal consistency across various Arabic-speaking populations (Sawaya et al., 2016; AlHadi et al., 2017). In this study, the scale demonstrated high reliability with a Cronbach’s alpha of 0.81.

### 2.7 Statistical analyses

All analyses were conducted using RStudio 4.4.2. First, descriptive statistics were calculated individually across all sociodemographic and clinical variables for each group. Response options for some questions in the original questionnaire were dichotomized or aggregated to create the new variable levels for some sociodemographic variables. For educational background, “low education” included the responses “no graduation” and “middle-school graduation,” while “high education” was made up of the responses “high school degree” and “university degree.” Regarding the kind of employment, "unemployed" included the responses “unemployed”, “in education”, “student”, “pensioned”, “housekeeper” and “other”; "employed was made up of the responses “employed” and “self-employed”. For family status, “in a relationship” included the categories “in a relationship”, “married” and “long-term partnership” and “not in a relationship” included “single”, “divorced” and “widowed”. Next, mean and standard deviation values were computed for metric parameters and absolute values and percentages for categorical variables. Next, for each variable, group differences were investigated for all possible comparisons as pairwise contrasts (RSG vs. SRS, RST vs. SRS, RSG vs. RST) using chi-square tests for categorical and *t*-tests for metric variables. These comparisons served two purposes: 1) between-group differences in sociodemographic parameters suggest using this parameter as a covariate in mediation models; 2) between-group comparisons in clinical variables - i.e. PTSD and depression - were performed to examine the first hypothesis relating to differences in symptom load between the two Syrian international refugee groups and the Syrian residents group.

Next, a set of analyses were performed to answer the mediation hypothesis. First, zero-order associations were computed for all variables for each comparison, resulting in three matrices of zero-order associations. Importantly, even in the absence of significant associations between two variables that were supposed to function as the predictor and the outcome in a given mediation model, the mediation was still examined since the indirect effect in mediation is still considered relevant even in the absence of such direct effects (Rucker et al., 2011; Zhao et al., 2010). Scatter plots and histograms were visually examined for each comparison and outcome separately to assess normality, linearity assumptions as well as outliers, and variance inflation factors were computed to rule out collinearity.

Six mediation analyses were computed as pairwise contrasts to account for each combination of group comparison (RSG vs. SRS, RST vs. SRS, RSG vs. RST) and outcome (depression and PTSD) using the lavaan package (Rosseel, 2012) and recent guidelines for mediation reporting were followed (Lee et al., 2021). For each model, grouping was defined as the predictor, the number of traumatic event types as the mediator and depression or PTSD scores as outcomes. Age, gender, family status, employment, and education were included as covariates in each model. Bootstrapping with 1000 estimates was employed to compute confidence intervals and inferential statistics for each model. For all analyses, an α level below 0.05 was considered significant.

Notably, Oaxaca-Blinder decompositions were performed to corroborate results from the mediation analysis (Hlavac, 2014). This set of analysis allows determining which part of the differences in the outcome variable is associated with being a refugee and which part is associated with other variables (e.g. sociodemographic parameters or trauma exposure). To this end, for each comparison (SRS vs. RSG, SRS vs. RST, RST vs. RSG) and each outcome (PCL-5, PHQ-9), a threefold Oaxaca-Blinder decomposition was computed as a pairwise contrast, with the given outcome modelled as a function of trauma exposure, employment status, education, age, family status, and gender, stratified by group membership. For models involving the Syrian residents group (SRS), SRS was used as the reference group and RST otherwise. Standard errors were obtained using 1000 bootstrapped computations. Three additional models, for each comparison group, were computed using trauma exposure as the outcome and the sociodemographic variables as predictors. The analysis rationale and a summary of results are detailed in the Supplementary Material (see S1-S9).

## 3 Results

### 3.1 Sociodemographic between-group comparisons

The three groups differed along several sociodemographic dimensions (see Table 1). The mean age was *M* = 25 years (*SD* = 7.9), *M* = 29 years (*SD* = 7.5), and *M* = 26 years (*SD* = 6.6) for SRS, RSG and RST, respectively. Significant age differences were found between SRS and RSG (*t* = −5.11, *p* < 0.001) and between RSG and RST (*t* = 4.51, *p* < 0.001), whereas the difference between SRS and RST was not significant (*t* = −0.82, *p* = 0.41). The sample was predominantly female in the SRS (59 %) and the RSG (57 %) group, with no significant difference between them (*t* = 0.16, *p* = 0.71). In contrast, the RST group had a lower proportion of female participants (43 %), and differed significantly from both the SRS (*t* = 10.68, *p* < 0.001) and RSG group (*t* = 8.17, *p* < 0.01). A significantly greater proportion of RSG participants (47 %) reported being in a relationship compared to SRS (30 %, *t* = 13.79, *p* < 0.001). The proportion of RST participants in a relationship (40 %) was also significantly greater than in SRS (*t* = 4.44, *p* < 0.04), but did not differ significantly from RSG (*t* = 1.74, *p* = 0.21). Employment rates were comparable between SRS (22 %) and RSG (24 %, *t* = 0.10, *p* = 0.80), while RST participants were more often employed (32 %), differing significantly from SRS (*t* = 4.66, *p* = 0.03), but not from RSG (*t* = 3.25, *p* = 0.07). Regarding education, significantly fewer RSG participants (78 %) reported a high level of education compared to SRS (86 %; *t* = 5.91, *p* = 0.015), while the education level of RST (86 %) was also significantly higher than in RSG (*t* = 5.24, *p* = 0.02), but did not differ from SRS (*t* = 0.00, *p* > 0.9).

**Table 1:** Sociodemographic data split for RSG, RST, and SRS.

				SRS vs. RSG		SRS vs. RST		RSG vs. RST	
Variable	SRS, *N* = 236^a^	RSG, *N* = 254^a^	RST, *N* = 199^a^	test stat^b^	p-value	test stat^b^	p-value	test stat^b^	p-value
Age	25.5 (7.9)	29.0 (7.5)	26.0 (6.6)	**−5.11**	**<0.001**	−0.82	0.4	**4.51**	**<0.001**
Gender				0.16	0.7	**10.68**	**0.001**	**8.17**	**0.004**
Female	139 (59 %)	144 (57 %)	85 (43 %)						
Male	97 (41 %)	110 (43 %)	114 (57 %)						
Family status^e^				**13.79**	**<0.001**	**4.44**	**0.035**	1.74	0.2
Not in relationship^e^	165 (70 %)	135 (53 %)	119 (60 %)						
In relationship^e^	71 (30 %)	119 (47 %)	80 (40 %)						
Employment^d^				0.10	0.8	**4.66**	**0.031**	3.25	0.072
Unemployed^d^	184 (78 %)	194 (76 %)	136 (68 %)						
Employed^d^	52 (22 %)	60 (24 %)	63 (32 %)						
Education^c^				**5.91**	**0.015**	0.00	>0.9	**5.24**	**0.022**
Low education^c^	32 (14 %)	57 (22 %)	27 (14 %)						
High education^c^	204 (86 %)	197 (78 %)	172 (86 %)						
Number of traumatic event types	4.6 (3.7)	6.9 (4.7)	6.7 (4.8)	**−5.94**	**<0.001**	**−4.87**	**<0.001**	0.49	0.6
Depression score	17.0 (5.6)	17.1 (5.6)	16.4 (6.1)	−0.11	>0.9	1.02	0.3	1.14	0.3
PTSD score	43.2 (16.4)	44.7 (14.5)	41.1 (16.2)	−1.12	0.3	1.30	0.2	**2.47**	**0.014**
Depression diagnosis	213 (90 %)	230 (91 %)	172 (86 %)	0.00	>0.9	1.20	0.3	1.50	0.2
PTSD diagnosis	176 (75 %)	205 (81 %)	144 (72 %)	2.32	0.13	0.17	0.7	**3.94**	**0.047**

*Note.* SRS = Syrians residing in Syria, RSG = Refugees from Syria resettled in Germany, RST = Refugees from Syria resettled in Turkey. Test stat = test statistic for a given test. Significant test statistics and p-values are marked in bold.

a n ( %); Mean (SD);.

b X^2^; t;.

c Low education includes “no graduation” and “middle-school graduation”; High education includes “high school degree” and “university degree”;.

d Unemployed includes “unemployed”, “in education”, “student”, “pensioned”, “housekeeper” and “other”; Employed includes “employed” and “self-employed”;.

e Not in relationship includes “single”, “divorced” and “widowed”; In a relationship includes “in a relationship”, “married” and “long-term partnership”.

### 3.2 Clinical between-group comparisons

With respect to the first research question – between-group comparisons regarding symptom load, prevalence rates, and exposure to traumatic events – relatively few differences emerged (Table 1). Participants in the RSG (*M* = 6.9, *SD* = 4.7) and RST (*M* = 6.7, *SD* = 4.8) group reported more traumatic event types than participants in the SRS group (*M* = 4.6, *SD* = 3.7), with both significantly different from SRS (SRS vs. RSG: *t* = −5.94, *p* < 0.001; SRS vs. RST: *t* = −4.87, *p* < 0.001), whereas no significant difference was found between RSG and RST (*t* = 0.49, *p* = 0.6). Depressive symptoms were similar across groups. Mean depression scores were *M* = 17.0 (*SD* = 5.6) for SRS, *M* = 17.1 (*SD* = 5.6) for RSG, and *M* = 16.4 (*SD* = 6.1) for RST, with no significant differences between SRS and RSG (*t* = −0.11, *p* > 0.9), SRS and RST (*t* = 1.02, *p* = 0.30), or RSG and RST (*t* = 1.14, *p* = 0.3). This was also reflected in prevalence rates for depression, which were 90 % for SRS, 91 % for RSG, and 86 % for RST meeting the cut-off for probable depression. No significant differences were found between any of the groups (SRS vs. RSG: *t* = 0.00, *p* > 0.9; SRS vs. RST: *t* = 1.20, *p* = 0.30; RSG vs. RST: *t* = 1.50, *p* = 0.20). Similarly, PTSD symptom severity did not significantly differ between SRS (*M* = 43.2, *SD* = 16.4) and RSG (*M* = 44.7, *SD* = 14.5, *t* = −1.12, *p* = 0.3) or between SRS and RST (*M* = 41.1, *SD* = 16.2, *t* = 1.30, *p* = 0.20). However, RSG participants reported significantly higher PTSD scores compared to RST (*t* = 2.47, *p* = 0.02). The prevalence rate of probable PTSD diagnosis was high across all groups, with 75 % for SRS, 81 % for RSG, and 72 % for RST. No significant differences between SRS and RSG (*t* = 2.32, *p* = 0.13) or between SRS and RST (*t* = 0.17, *p* = 0.70) were found, but RSG participants had a significantly higher probable PTSD prevalence than RST (*t* = 3.94, *p* = 0.04).

### 3.3 Zero-order correlations

Zero-order correlations were calculated for all variable pairs per comparison (i.e., RSG vs. SRS, RST vs. SRS, RSG vs. RST); for brevity, only correlation results between predictor, mediator, and outcome are mentioned for each comparison. Correlations for the RSG vs. SRS comparison are shown in Table 2.

**Table 2:** Zero-order associations for RSG-SRS comparison.

Variable	1	2	3	4	5	6	7	8	9
1. PTSD score	-								
2. Depression score	*t*=12.71, *r*=0.50***	-							
3. Number of traumatic event types	*t*=3.99, *r*=0.18***	*t* = 1.51, *r* = 0.07	-						
4. Group	*t*=−1.12, *d*=−0.10	*t*=−0.11, *d*=−0.01	*t*=−5.94, *d*=−0.53***	-					
5. Employment	*t*=−1.14, *d*=−0.11	*t*=2.40, *d*=0.26*	*t*=−0.32, *d*=−0.04	χ²=0.17, φ=0.02	-				
6. Education	*t* = 1.55, *d* = 0.18	*t*=2.35, *d*=0.23*	*t* = 1.82, *d* = 0.24	χ²=6.49, φ=0.12*	χ²=4.20, φ=0.09*	-			
7. Age	*t*=2.11, *r*=0.09*	*t* = 0.46, *r* = 0.02	*t*=3.41, *r*=0.15***	*t*=−5.11, *d*=−0.46***	*t*=−7.32, *d*=−0.75***	*t*=−2.76, *d*=−0.33**	-		
8. Family status	*t*=−0.02, *d*=−0.00	*t* = 1.22, *d* = 0.11	*t* = 1.86, *d* = 0.17	χ²=14.48, φ=0.17***	χ²=4.47, φ=0.10*	χ²=0.13, φ=0.02	*t*=−6.43, *d*=−0.65***	-	
9. Gender	*t* = 0.72, *d* = 0.07	*t* = 0.36, *d* = 0.03	*t*=−2.07, *d*=−0.20*	χ²=0.24, φ=0.02	χ²=11.67, φ=0.15***	χ²=0.32, φ=0.03	*t*=−3.31, *d*=−0.30**	χ²=3.71, φ=0.09	-

*Note.* SRS = Syrians residing in Syria, RSG = Refugees from Syria resettled in Germany, PTSD = Posttraumatic stress disorder. Pearson’s correlations were calculated for associations of two numeric variables; Welch’s tests and a corresponding Cohen’s d were computed if one variable was binary and the other numeric, and Chi-square tests and Phi were calculated in case both variables were binary. Significance levels: * *p* ≤ 0.05, ** *p* ≤ 0.01, *** *p* ≤ 0.001.

PTSD scores correlated significantly with the number of traumatic events(*r* = 0.18, *p* < 0.001) but not with group membership (*t*(469.77) = 1.12, *p* = 0.26). Depression scores were not significantly associated with either the number of traumatic events (*r* = 0.07, *p* = 0.13) or group membership (*t*(484.95) = −0.11, *p* = 0.91). The number of traumatic events was significantly different between groups (*t*(477.83) = −5.94, *p* < 0.001).

For the RST vs. SRS comparison (Table 3), PTSD scores were significantly correlated with the number of traumatic events (*r* = 0.24, *p* < 0.001) but not with group membership (*t*(422.24) = 1.30, *p* = 0.19). Depression scores were also significantly associated with the number of traumatic events (*r* = 0.11, *p* < 0.05) but not with group membership (*t*(408.58) = 1.02, *p* = 0.31). Moreover, the number of traumatic events was significantly associated with group membership (*t*(370) = −4.87, *p* < 0.001).

**Table 3:** Zero-order associations for RST-SRS comparisons.

Variable	1	2	3	4	5	6	7	8	9
1. PTSD score	-								
2. Depression score	*t*=12.17, *r*=0.50***	-							
3. Number of traumatic event types	*t*=5.25, *r*=0.24***	*t*=2.35, *r*=0.11*	-						
4. Group	*t* = 1.30, *d* = 0.13	*t* = 1.02, *d* = 0.10	*t*=−4.87, *d*=−0.48***	-					
5. Employment	*t*=−0.47, *d*=−0.05	*t* = 0.84, *d* = 0.09	*t*=−2.17, *d*=−0.26*	χ²=5.14, φ=0.11*	-				
6. Education	*t* = 0.50, *d* = 0.07	*t*=2.33, *d*=0.30*	*t* = 1.80, *d* = 0.32	χ²=0.00, φ=0.00	χ²=0.26, φ=0.02	-			
7. Age	*t* = 0.68, *r* = 0.03	*t* = 0.02, *r* = 0.00	*t* = 1.69, *r* = 0.08	*t*=−0.82, *d*=−0.08	*t*=−7.80, *d*=−0.87***	*t*=−2.09, *d*=−0.28*	-		
8. Family status	*t* = 0.50, *d* = 0.05	*t*=−0.55, *d*=−0.06	*t*=−0.16, *d*=−0.02	χ²=4.88, φ=0.11*	χ²=7.61, φ=0.13**	χ²=1.77, φ=0.06	*t*=−6.43, *d*=−0.74***	-	
9. Gender	*t* = 0.44, *d* = 0.04	*t* = 0.95, *d* = 0.09	*t*=−3.72, *d*=−0.36***	χ²=11.32, φ=0.16***	χ²=32.53, φ=0.27***	χ²=5.52, φ=0.11*	*t*=−3.79, *d*=−0.37***	χ²=0.13, φ=0.02	-

*Note.* SRS = Syrians residing in Syria, RST = Refugees from Syria resettled in Turkey, PTSD = Posttraumatic stress disorder. Pearson’s correlations were calculated for associations of two numeric variables; Welch’s tests and a corresponding Cohen’s d were computed if one variable was binary and the other numeric, and Chi-square tests and Phi were calculated in case both variables were binary. Significance levels: * *p* ≤ 0.05, ** *p* ≤ 0.01, *** *p* ≤ 0.001.

Lastly, correlations were also computed for the RSG vs. RST comparison (Table 4). PTSD scores were significantly correlated with both the number of traumatic events (*r* = 0.27, *p* < 0.001) and group membership (*t*(400.41) = 2.47, *p* < 0.05). Depression scores were also significantly correlated with the number of traumatic events (*r* = 0.16, *p* < 0.001) but not with group membership (*t*(408.27) = 1.14, *p* = 0.26). The number of traumatic events was not significantly correlated with group membership (*t*(419.06) = 0.49, *p* = 0.63).

**Table 4:** Zero-order correlations for RSG-RST comparison.

Variable	1	2	3	4	5	6	7	8	9
1. PTSD score	-								
2. Depression score	*t*=12.50, *r*=0.51***	-							
3. Number of traumatic event types	*t*=6.01, *r*=0.27***	*t*=3.44, *r*=0.16***	-						
4. Group	*t*=2.47, *d*=0.24*	*t* = 1.14, *d* = 0.11	*t* = 0.49, *d* = 0.05	-					
5. Employment	*t* = 0.46, *d* = 0.05	*t* = 1.74, *d* = 0.19	*t*=−0.19, *d*=−0.02	χ²=3.64, φ=0.09	-				
6. Education	*t*=2.04, *d*=0.23*	*t*=2.95, *d*=0.31**	*t* = 0.77, *d* = 0.09	χ²=5.82, φ=0.11*	χ²=2.49, φ=0.07	-			
7. Age	*t* = 1.77, *r* = 0.08	*t*=−0.08, *r*=−0.00	*t*=2.04, *r*=0.10*	*t*=4.51, *d*=0.42***	*t*=−5.90, *d*=−0.57***	*t*=−3.69, *d*=−0.46***	-		
8. Family status	*t*=−1.06, *d*=−0.10	*t*=−0.07, *d*=−0.01	*t* = 1.77, *d* = 0.17	χ²=2.00, φ=0.07	χ²=10.15, φ=0.15**	χ²=0.21, φ=0.02	*t*=−6.60, *d*=−0.65***	-	
9. Gender	*t* = 1.01, *d* = 0.09	*t* = 0.33, *d* = 0.03	*t*=−3.05, *d*=−0.29**	χ²=8.72, φ=0.14**	χ²=26.10, φ=0.24***	χ²=0.13, φ=0.02	*t*=−3.20, *d*=−0.30**	χ²=4.66, φ=0.10*	-

*Note.* RSG = Refugees from Syria resettled in Germany, RST = Refugees from Syria resettled in Turkey, PTSD = Posttraumatic stress disorder. Pearson’s correlations were calculated for associations of two numeric variables; Welch’s tests and a corresponding Cohen’s d were computed if one variable was binary and the other numeric, and Chi-square tests and Phi were calculated in case both variables were binary. Significance levels: * *p* ≤ 0.05, ** *p* ≤ 0.01, *** *p* ≤ 0.001.

### 3.4 Mediation analysis

As mentioned above, mediation analyses were conducted separately for each comparison, resulting in six models. Specifically, grouping was defined as a predictor, the number of traumatic event types as a mediator, and depression/PTSD score as the outcome, with age, gender, family status, education and employment used as covariates. Standardized estimates and 95 % confidence intervals, and the standard error pertaining to bootstrapped total, direct, and indirect effects are reported (Table 5). First, the RSG vs. SRS comparison was examined. Regarding depression symptoms, the indirect effect from grouping through the number of traumatic event types to depression score was non-significant (*β* = 0.012, 95 % CI [−0.010, 0.034], *p* = 0.274), nor were the total (*β* = −0.012, 95 % CI [−0.104, 0.080], *p* = 0.79) or direct effects (*β* = −0.024, 95 % CI [−0.118, 0.069], *p* = 0.61). For PTSD, the indirect effect was significant (*β* = 0.040, 95 % CI [0.015, 0.065], *p* = 0.002). This indicates partial mediation, given both the total effect (*β* = 0.024, 95 % CI [−0.112, 0.081], *p* = 0.62) and the direct effects (*β* = −0.016, 95 % CI [−0.072, 0.120], *p* = 0.75) were not significant, i.e. the number of traumatic event types experienced explains differences in posttraumatic symptom severity when comparing Syrian refugees residing in Germany to Syrians residing in Syria.

**Table 5:** Mediation results per comparison, per outcome.

	Standardized estimate β (95 % CI)	S.E.	p-value
**SRS vs. RSG**			
*Depression*			
Total effect	−0.012 (−0.104 – 0.080)	0.047	0.797
Direct effect	−0.024 (−0.118 – 0.069)	0.048	0.609
Indirect effect	0.012 (−0.010 – 0.034)	0.011	0.274
*PTSD*			
Total effect	0.024 (−0.112 – 0.081)	0.049	0.618
Direct effect	−0.016 (−0.072 – 0.120)	0.049	0.751
Indirect effect	0.04 (0.015 – 0.065)**	**0.013**	**0.002**
**SRS vs. RST**			
*Depression*			
Total effect	−0.041 (−0.130 – 0.047)	0.045	0.362
Direct effect	−0.069 (−0.160 – 0.021)	0.046	0.134
Indirect effect	0.028 (0.003 – 0.053)*	**0.013**	**0.028**
*PTSD*			
Total effect	−0.058 (−0.153 – 0.038)	0.049	0.235
Direct effect	−0.117 (−0.209 – −0.025)**	**0.047**	**0.013**
Indirect effect	0.059 (0.028 – 0.090)***	**0.016**	**< 0.001**
**RSG vs. RST**			
*Depression*			
Total effect	−0.028 (−0.125 – 0.070)	0.050	0.577
Direct effect	−0.026 (−0.122 – 0.071)	0.049	0.605
Indirect effect	−0.002 (−0.018 – 0.014)	0.008	0.790
*PTSD*			
Total effect	−0.078 (−0.169 – 0.012)	0.046	0.088
Direct effect	−0.075 (−0.162 – 0.013)	0.045	0.095
Indirect effect	−0.004 (−0.031 – 0.024)	0.014	0.791

*Note.* SRS = Syrians residing in Syria, RSG = Refugees from Syria resettled in Germany, RST = Refugees from Syria resettled in Turkey, PTSD = Posttraumatic stress disorder. CI = Confidence interval, S.E. = Standard error. Significance levels: * *p* ≤ 0.05, ** *p* ≤ 0.01, *** *p* ≤ 0.001.

Next, mediation effects for the RST vs. SRS comparison were investigated. In contrast to the RSG vs. SRS model, the indirect effect for depression was significant (*β* = 0.028, 95 % CI [0.003, 0.053], *p* = 0.03), suggesting partial mediation, given the non-significant total (*β* = −0.041, 95 % CI [−0.130, 0.047], *p* = 0.36) and direct effects (*β* = −0.069, 95 % CI [−0.160, 0.021], *p* = 0.13). For PTSD, both the indirect (*β* = 0.059, 95 % CI [0.028, 0.090], *p* < 0.001) and direct effects (*β* = −0.117, 95 % CI [−0.209, −0.025], *p* = 0.01) were significant, indicating full mediation, given the non-significant total effect (*β* = −0.058, 95 % CI [−0.153, 0.038], *p* = 0.23). Thus, for both depression and PTSD, the number of traumatic event types experienced explains differences in symptom load between Syrians who fled to Turkey compared to Syrians who remained in Syria.

Lastly, RSG and RST were compared within the mediation framework. For depression, there was no indirect effect (*β* = −0.002, 95 % CI [−0.018, 0.014], *p* = 0.79), as well as no total (*β* = −0.028, 95 % CI [−0.125, 0.070], *p* = 0.58) or direct effects (*β* = −0.026, 95 % CI [−0.122, 0.071], *p* = 0.61). The same was true for PTSD, where the indirect (*β* = −0.004, 95 % CI [−0.031, 0.024], *p* = 0.791), direct (*β* = −0.075, 95 % CI [−0.162, 0.013], *p* = 0.09) and total effects (*β* = −0.078, 95 % CI [−0.169, 0.012], *p* = 0.09) were all non significant. Therefore, the number of traumatic event types experienced does not explain differences in either depression or PTSD symptoms when comparing refugees who fled to Germany to those who fled to Turkey.

Notably, Oaxaca-Blinder decompositions supported these results: Differences in symptom severity between Syrian international refugees and Syrian residents were associated with differences in predictor variable distributions (endowments), with the number of traumatic events being the most important predictor across multiple models. This was true for both group comparisons regarding trauma symptoms, and for SRS vs. RST regarding symptoms of depression. Moreover, differences in the number of traumatic events were mostly associated with being a refugee versus being a Syrian resident (coefficients) and not to other sociodemographic factors. Methodology and a summary of the results are summarized in greater detail in the Supplementary Material (see S1-S9).

Overall, significant mediation effects of the number of traumatic event types on PTSD and depression symptoms were only observed for the RSG/RST groups in comparison to SRS but not between RSG and RST.

## 4 Discussion

### 4.1 Main results

The present research investigates differences in depression and PTSD prevalence rates and symptom severity between traumatized, treatment-seeking Syrians who either fled to Germany or Turkey or remained in Syria. Previous research has endeavoured to gauge the impact of being a refugee on mental health, mostly by making cross-study comparisons of refugees resettled in high-income, Western countries to non-refugee populations resettled in those counties or living in common regions of origin of refugees, such as the SWANA region (UNHCR. Global Trends Report 2022 2023). This approach comes with the caveat that the groups of comparison not only differ regarding not/being a refugee but also with respect to either country of origin or country of residence, thus confounding between-group differences. The present design addresses some of this variability by examining a single group (Syrians) across different countries of residence (Syrian residents in Syria, Syrian refugees in Germany, Syrian refugees in Turkey).

We first hypothesized that Syrian refugees in both Germany and Turkey would display higher depression and PTSD prevalence rates and severities than Syrian residents in Syria. However, the present results show no differences between either Syrian international refugee group compared to the Syrian residents group in symptoms for both disorders, even though the number of traumatic events reported was significantly higher in the refugee groups compared to the group of Syrian participants still living in Syria. Instead, we found a significantly higher prevalence rate and symptom severity regarding PTSD for Syrian refugees in Turkey compared to Syrian refugees in Germany. A possible explanation for this pattern of results is that all participants included in the current study were treatment-seeking, and, more specifically, registered for an intervention targeting PTSD and depression. Treatment-seeking populations may have a higher symptom load than non-treatment-seeking populations, prompting them to reach out for help (Hlavac, 2014). In the present sample, in particular, prevalence rates were remarkably high, with at least 86 % of participants showing clinically relevant symptoms of depression and 72 % scoring above the cut-off for probable PTSD in each of the groups, constituting a potential ceiling effect. These results are notably higher than previous findings on non-treatment-seeking Syrian refugees, showing the highest rates with 28.7 % for depression and 25.3 % for PTSD in Germany (Borho et al., 2020; Schoenberger et al., 2024) and 47.7 % for depression and 41.6 % for PTSD in Turkey (Kaya et al., 2019; Kurt et al., 2021). In the present study, this potential ceiling effect may have been compounded by the fact that all participants in the sample had experienced at least one traumatic event. Thus, the impact of (not) being an international refugee on prevalence rates may have been too small to detect. Interestingly, refugees who resettled in Germany did show higher PTSD symptom load and PTSD prevalence than those who fled to Turkey. Importantly, though, this effect disappeared when accounting for between-group differences in sociodemographic variables and different types of trauma experienced in the mediation model, indicating that this effect is more likely due to sample composition (i.e. differences in the distribution of gender, age, marital status, etc.). Overall, these results indicate a notably high depression and PTSD symptom load among treatment-seeking people from Syria, however independent from being international refugees.

Beyond prevalence rates of PTSD and depression, analyses showed that Syrian refugees outside of Syria in this sample did experience significantly more traumatic event types than Syrian residents, with an average of approximately seven event types for both refugee groups and about four event types for Syrian residents. In comparison, Tinghög and colleagues (2017) reported four different event types in Syrian refugees pre-flight and two peri‑flight events (Tinghög et al., 2017). However, Tinghög et al.’s findings cannot be compared directly to the present findings because in the present study no distinction between the time of the trauma experience is possible and in addition, Tinghög and colleagues assessed non-treatment-seeking Syrian refugees residing in Sweden. In a registry-based study in Germany (Georgiadou et al., 2018), a mean of 2.2 traumatic events was found. While the present study reports a higher average, this may again be due to a different questionnaire used and the fact that all participants had registered for a psychotherapy targeting PTSD and depression. Nevertheless, the present study allows a comparison of the number of traumatic events experienced between Syrian refugees outside of Syria and Syrian residents within one methodological framework. Possible explanations might be that participants who had experienced a higher number of traumatic events were more likely to have to flee their home country or experienced more diverse traumatic event types during flight and afterwards. While longitudinal study designs may be best suited to resolve this question, the present study indicates differences in trauma types exposure between Syrian residents and Syrian international refugees, independent of the host country.

The second hypothesis postulated that trauma exposure explains the association between group membership and PTSD/depression symptom load. While there was no direct association between group membership and symptom load for either outcome, there may still be an indirect association through a third variable (Rucker et al., 2011; Zhao et al., 2010), namely, the number of traumatic event types experienced. The present study found that the number of traumatic event types mediated the relationship between being a Syrian refugee in Germany compared to residing in Syria with respect to PTSD symptoms. However, there was no mediation for this group comparison regarding depressive symptoms. For Syrian refugees in Turkey compared to Syrian participants residing in Syria, the number of traumatic event types mediated the associations to both PTSD and depressive symptoms. In other words, these findings suggest that – in the three cases where there was mediation – posttraumatic symptoms did not arise due to being an international refugee but might arise through the additional trauma type exposure associated with being a refugee. These findings are consistent with theories postulating that posttraumatic symptoms might sometimes only emerge later - potentially in safer environments - in the context of continuous trauma (Slewa-Younan et al., 2014; Pat-Horenczyk and Schiff, 2019). In a similar vein, refugees’ posttraumatic symptoms may mainly arise once they have arrived in a host country and the traumatizing context - i.e. traumatic events in the country of origin and during flight - is left behind. The present mediation findings are consistent with this idea. Notably, there was no mediating effect of the number of traumatic event types for either outcome (depression/PTSD) when comparing between refugee groups. This suggests that the described effect might not be specific to a host country. Most host countries, even or especially in Europe, do not constitute safe environments for refugees because of experiences of racial harassment and xenophobia, which have been shown to predict mental illness (Stevens et al., 2013; Suleman et al., 2018). Other factors, such as struggling to adapt to a new cultural and political environment, also have a detrimental psychological impact (Choy et al., 2021). It can’t be ruled out that these factors or other context factors contributed to psychopathological symptoms in participants, as they were not assessed in the current study. Moreover, given the cross-sectional design, the exact time of symptom development (i.e. pre-, peri‑ or post-flight) can also not be detected. Nevertheless, the mediation results suggest that the experience of a larger number of trauma types is related to psychopathological symptoms present in international refugees.

### 4.2 Limitations

While the analyses present unique results comparing Syrian residents and Syrian refugees across different residence countries, there are caveats to the present approach. First, the results are not necessarily generalizable across refugee groups from other countries, even within the SWANA region. For instance, cultural and socio-political influences in different refugees’ origin countries may influence how the living conditions of refugees manifest in mental health outcomes. A similar criticism applies to the host country. The present study includes two host countries to reduce some of the variability that may be attributable to this country variable, and concludes that the results for severity of PTSD and depression remain broadly similar across these countries. However, including further host countries may lead to different results due to different flight experiences or living conditions pre- and post-flight. Additionally, while the study does differentiate between Syrian residents and international refugees from Syria, there was no information recorded to allow distinguishing between internally displaced persons (IDPs) and non-displaced persons within Syria. Thus, the present design cannot ascertain whether Syrians within Syria have experienced some form of displacement, even if not across state borders. The experience of being internally displaced may influence mental health outcomes in ways both similar to and different from refugees, potentially impacting the results presented here (e.g. by raising the average mental health burden in the SRS group). Building on the idea of comparisons of IDPs and non-IDPs in Syria, an alternative design would compare Syrian refugees and non-refugees within the same host country (e.g., in Germany). Although this approach may introduce other biases (e.g., selection effects or differences in social benefits), it would better control for contextual factors and offer an additional perspective on the mental health impact of being a refugee. Future research should pursue such designs and extend the present approach to additional countries of origin and refuge. Moreover, the treatment-seeking aspect of this study’s sample limits the scope of interpretations. As mentioned before, the treatment-seeking sample may have created a ceiling effect, whereby participants—no matter if they are refugees or not— experience high symptom severity, minimizing between-group differences. Thus, the present study design may miss between-group differences in mental health between Syrian international refugees and Syrian residents. Additionally, this study employs a cross-sectional design, with the study outcome based on self-report screening measures. Due to the cross-sectional nature of data analysed, the causality of the reported associations cannot be determined, as both predictors and outcomes were measured at the same time. Further, some of the predictors, such as number of traumatic event types, rely on recollections of past events, making them amenable to memory biases. Finally, using self-report screening measures as outcomes might lead to biases. For instance, participants may pick responses to self-report questionnaires in a socially desirable way (Schouler-Ocak and Moran, 2023). Additionally, while screening measures are often less time-intensive than clinician-based diagnostic interviews, they lack accuracy in diagnosing psychopathology (Brenner and DeLamater, 2016; Sheldrick et al., 2015). Given that the present results are also based on screening measures, they must be interpreted with caution and ideally be validated using clinician-based interviews in future studies.

### 4.3 Conclusions

The present study investigates differences in mental health outcomes among a treatment-seeking population from Syria, who either remained in Syria or resettled in Germany or Turkey. The results reveal no differences in depression and PTSD prevalence rates between Syrian international refugees and Syrian residents; however, differences are found regarding the number of traumatic events experienced which in turn mediate the association between being a Syrian international refugee vs. Syrian resident and mental health outcomes. This study design allows linking these differences to being a refugee outside of Syria, while accounting for some of the confounding influences of between-group differences in country of origin or residence. Results must be interpreted with caution, however, given the treatment-seeking nature of the sample and the cross-sectional study design. Nevertheless, these findings underscore the need for broad and efficient psychosocial support for Syrians independent of their current place of residence, given that people in Syria have gone through over a decade of war, internal displacement and instability, partly still lasting.

## Abbreviations

SRS: Syrians residing in Syria

RSG: Refugees from Syria living in Germany

RST: Refugees from Syria living in Turkey

PTSD: Post-traumatic stress disorder

SWANA: Southwest Asia and North Africa

RTHC: Refugee trauma history checklist

HTQ: Harvard Trauma Questionnaire

PDS: Posttraumatic Diagnostic Scale

LEC-5: Life Events Checklist for DSM-5

PCL-5: PTSD Checklist for DSM-5

PHQ-9: Patient Health Questionnaire-9

## Ethics approval and consent to participate

The studies involving human participants were reviewed and approved by the ethics committee at Freie Universität Berlin (126/2016 and 185/2018). The participants provided written informed consent to participate in this study.

## Consent for publication

Informed consent was obtained from all subjects involved in the study.

## Availability of data and materials

Data cannot be shared publicly because the dataset contains sensitive information that 1) could be misused for political purposes, 2) describe individuals who may be experiencing political persecution, and are therefore at risk, 3) describe individuals who have experienced sexual violence within the family or within the community and live in regions where exposure to sexual violence can have serious consequences for the survivors (e.g. prison sentences, punishment by family, exclusion from society), and are therefore at risk of harm, and 4) describe clinical data with potential patients for treatment of posttraumatic stress and depression. Data are available from the center ÜBERLEBEN (contact via y.nesterko@ueberleben.org) for researchers who meet the criteria for access to confidential data, particularly if the purpose of the study is made clear and it is clear which working group will receive the data.

## Funding

This project was funded by Misereor e.V.. The funders had no role in the study design, data collection, analysis, interpretation, or in the decision to submit the article.

## CRediT authorship contribution statement

**Max Bringmann:** Writing – review & editing, Writing – original draft, Methodology, Formal analysis. **Maya Böhm:** Writing – review & editing. **Freya Specht:** Writing – review & editing. **Max Vöhringer:** Writing – review & editing. **Majdy Aldoibal:** Writing – review & editing. **Christine Knaevelsrud:** Writing – review & editing, Conceptualization. **Birgit Wagner:** Writing – review & editing, Conceptualization. **Maria Böttche:** Writing – review & editing, Supervision, Conceptualization. **Yuriy Nesterko:** Writing – review & editing, Supervision, Formal analysis, Conceptualization.

## Declaration of competing interest

The authors declare that they have no known competing financial interests or personal relationships that could have appeared to influence the work reported in this paper.

## Appendix Supplementary materials

Image, application 1
