# Supplementary material for: Does country of residence matter? A cross-sectional comparison of PTSD and depression among traumatized, treatment-seeking Syrians residing in Syria and Syrian refugees in Germany and Turkey
Source: J Migr Health. 2026 Jan 28;13:100399. doi: 10.1016/j.jmh.2026.100399 (PMC12914855; doi:10.1016/j.jmh.2026.100399)
Supplement: Supplementary file 1 [file mmc1.docx]

**Supplementary Material:** “Does country of residence matter? A cross-sectional comparison of PTSD and depression among traumatized, treatment-seeking Syrians residing in Syria and Syrian refugees in Germany and Turkey”

Oaxaca-Blinder decomposition - analysis rationale, procedure and summary of results

The Oaxaca-Blinder decomposition is a method that has commonly been applied in the field of labor market discrimination research across different forms of discrimination [1, 2]. The approach aims to explain differences in an outcome variable between two groups by splitting these differences up into several components [3]: The endowments component captures differences in the outcome variable due to differences in the distributions of predictors across the two groups. In the present study, a hypothetical example may be that differences in the gender distribution between a Syrian international refugee and a Syrian residents group explain part of the differences in depressive symptoms. The coefficients component captures differences in the outcome variable that are attributable to differences in predictor coefficients between the groups. For instance, age may be differently associated (more strongly, with a different polarity) with the depression outcome in a refugee compared to a Syrian residents group. Lastly, the interaction component represents the potential simultaneous occurrence of cross-group differences in coefficients and endowments. Notably, individual predictors can be examined for statistical significance for a given component. In the present example, trauma load may be a significant predictor of the endowments component, indicating that differences in the distribution of trauma exposure between groups explain differences in outcome. In this sense, the Oaxaca-Blinder decomposition offers an additional angle to the main analysis - i.e. the mediation models - by showing which part of the outcome difference is due to the distributional differences of predictors between groups (endowments) and which part is due to the groups (coefficients, i.e. the way the coefficients differ for each of them). To this end, for each comparison (SRS vs. RSG, SRS vs. RST, RST vs. RSG) and each outcome (PCL-5, PHQ-9), a threefold Oaxaca-Blinder decomposition was computed, with the given outcome modelled as a function of trauma exposure, employment status, education, age, family status, and gender, stratified by group membership. For models involving the Syrian residents group (SRS), SRS was used as the reference group and RST otherwise. Standard errors were obtained using 1000 bootstrapped computations. Three additional models, for each comparison group, were computed using trauma exposure as the outcome and the sociodemographic variables as predictors.

Results for these computations can be summarized as follows. For the Syrian international refugee vs. Syrian resident comparisons (SRS vs. RSG, SRS vs. RST), the models showed significant components only for the trauma outcomes. Specifically, the endowment components were significant for each model (SRS vs. RSG: *β* = -2.733, 95% CI [-4.250, -1.216], *p* < 0.001; SRS vs. RST: *β* = -2.251, 95% CI [-3.943, -0.558], *p* = 0.009), and, for each model, the trauma exposure predictor was the only significant predictor within the endowments component (SRS vs. RSG: *β* = -1.299, 95% CI [-2.217, -0.380], *p* = 0.006; SRS vs. RST: *β* = -2.661, 95% CI [-4.067, -1.255], *p* < 0.001). This indicates that differences in trauma symptom outcomes between these groups can be attributed to between-group differences in trauma exposure, consistent with mediation analysis results (see S1-S4 for all the results of the refugee vs. Syrian resident models). Additionally, for the trauma outcome, the endowment component was significant for the comparison of Syrian refugees in Germany versus Syrian refugees in Turkey (NSG vs. NST: *β* = -1.636, 95% CI [-2.934, -0.339], *p* < 0.013), though no single predictor was above-chance for the endowment component (see S5-S6).

Lastly, Oaxaca-Blinder decompositions were also calculated using trauma exposure as outcome, for all three comparisons separately and using only the sociodemographic variables from the previous models as predictors. Here, only the coefficient component was significant, and only for the models examining a Syrian international refugee versus Syrian resident comparison (SRS vs. RSG: *β* = -2.082, 95% CI [-2.944, -1.219], *p* < 0.001; SRS vs. RST: *β* = -2.007, 95% CI [-2.876, -1.139], *p* < 0.001), with no predictor showing an above-chance association with the outcome for this component in either model. This finding shows that the difference in trauma exposure can be attributed to the groups (Syrian refugee outside of Syria vs. Syrian resident) and cannot be explained by the sociodemographic variables used in the model. Thus, overall, the findings from the Oaxaca-Blinder decompositions corroborate the results from the mediation models, showing that differences between Syrian international refugee versus Syrian resident groups can be primarily explained by differences in trauma exposure. Notably, the result was only different for the depression symptoms model of the RST vs. SRS comparison, where a trauma exposure was a significant mediator for the mediation models, but where the endowment component was only marginally significant for the decomposition analysis (SRS vs. RST: *β* = -0.535, 95% CI [-1.146, 0.076], *p* = 0.086). Nevertheless, the trauma exposure predictor was the only significant predictor also for this model’s endowment component (*β* = -0.584, 95% CI [-1.080, -0.087], *p* = 0.021). Thus, the Oaxaca-Blinder decomposition for the most part corroborates the mediation model’s results also in this instance.

RSG vs. SRS - Oaxaca-Blinder decomposition results - trauma symptoms

PTSD severity was higher in the RSG group (n = 254, M = 44.75) compared with the SRS group (n = 236, M = 43.17), with a mean difference of Δ ≈ 1.58. Oaxaca–Blinder decomposition indicated that approximately 66% of this difference was explained by group differences in observed characteristics (endowments), 3% by differences in coefficients, and 31% by interaction effects.

**S1**

*Oaxaca-Blinder decomposition for SRS vs. RSG - PTSD outcome*

|  | **Standardized estimate β (95% CI)** | **S.E.** | **p-value** |
| --- | --- | --- | --- |
| *Decomposition* |  |  |  |
| Endowments | -2.733 (-4.250, -1.216) | 0.774 | 0.000 |
| Coefficients | -0.115 (-3.149, 2.919) | 1.548 | 0.941 |
| Interaction | 1.270 (-0.909, 3.449) | 1.112 | 0.253 |
| *Endowments (explained)* |  |  |  |
| Trauma exposure | -1.299 (-2.217, -0.380) | 0.468 | 0.006 |
| Employment: Employed | -0.006 (-0.166, 0.154) | 0.082 | 0.942 |
| Education: High | -0.422 (-0.900, 0.055) | 0.244 | 0.083 |
| Age | -0.837 (-1.795, 0.122) | 0.489 | 0.087 |
| Family status: In relationship | -0.221 (-0.886, 0.444) | 0.339 | 0.514 |
| Gender: Male | 0.052 (-0.231, 0.334) | 0.144 | 0.720 |
| *Coefficients (unexplained)* |  |  |  |
| Trauma exposure | 0.481 (-4.162, 5.124) | 2.369 | 0.839 |
| Employment: Employed | 0.509 (-0.971, 1.988) | 0.755 | 0.500 |
| Education: High | 3.235 (-3.163, 9.633) | 3.264 | 0.322 |
| Age | -3.386 (-14.791, 8.018) | 5.819 | 0.561 |
| Family status: In relationship | -1.930 (-4.794, 0.934) | 1.461 | 0.186 |
| Gender: Male | 0.316 (-2.246, 2.879) | 1.307 | 0.809 |

*Note.* SRS = Syrians residing in Syria, RSG = Refugees from Syria resettled in Germany, PTSD = Posttraumatic stress disorder. CI = Confidence interval, S.E. = Standard error. Significance levels: * *p* ≤ 0.05, ** *p* ≤ 0.01, *** *p* ≤ 0.001. SRS was used as the reference group.

RSG vs. SRS - Oaxaca-Blinder decomposition results - depressive symptoms

Depression severity was higher in the RSG group (n = 254, M = 17.08) compared with the SRS group (n = 236, M = 17.02), with a mean difference of Δ ≈ 0.06. Oaxaca–Blinder decomposition indicated that approximately 54% of this difference was explained by group differences in observed characteristics (endowments), 28% by differences in coefficients, and 18% by interaction effects.

**S2**

*Oaxaca-Blinder decomposition for SRS vs. RSG - depression outcome*

|  | **Standardized estimate β (95% CI)** | **S.E.** | **p-value** |
| --- | --- | --- | --- |
| *Decomposition* |  |  |  |
| Endowments | -0.366 (-0.926, 0.194) | 0.286 | 0.201 |
| Coefficients | 0.188 (-0.964, 1.341) | 0.588 | 0.749 |
| Interaction | 0.120 (-0.676, 0.915) | 0.406 | 0.768 |
| *Endowments (explained)* |  |  |  |
| Trauma exposure | -0.216 (-0.572, 0.139) | 0.181 | 0.232 |
| Employment: Employed | 0.029 (-0.128, 0.186) | 0.080 | 0.718 |
| Education: High | -0.116 (-0.273, 0.041) | 0.080 | 0.148 |
| Age | -0.170 (-0.478, 0.138) | 0.157 | 0.280 |
| Family status: In relationship | 0.109 (-0.145, 0.362) | 0.130 | 0.402 |
| Gender: Male | -0.001 (-0.074, 0.072) | 0.037 | 0.978 |
| *Coefficients (unexplained)* |  |  |  |
| Trauma exposure | -0.571 (-2.228, 1.086) | 0.845 | 0.499 |
| Employment: Employed | 0.144 (-0.462, 0.749) | 0.309 | 0.642 |
| Education: High | -0.055 (-1.955, 1.845) | 0.969 | 0.955 |
| Age | 0.732 (-2.997, 4.460) | 1.902 | 0.701 |
| Family status: In relationship | -0.064 (-1.109, 0.982) | 0.534 | 0.905 |
| Gender: Male | -0.285 (-1.183, 0.613) | 0.458 | 0.534 |

*Note.* SRS = Syrians residing in Syria, RSG = Refugees from Syria resettled in Germany. CI = Confidence interval, S.E. = Standard error. Significance levels: * *p* ≤ 0.05, ** *p* ≤ 0.01, *** *p* ≤ 0.001.

RST vs. SRS - Oaxaca-Blinder decomposition results - trauma symptoms

PTSD severity was higher in the RST group (n = 199, M = 41.13) compared with the SRS group (n = 236, M = 43.17), with a mean difference of Δ ≈ 2.04. Oaxaca–Blinder decomposition indicated that approximately 34% of this difference was explained by group differences in observed characteristics (endowments), 48% by differences in coefficients, and 18% by interaction effects.

**S3**

*Oaxaca-Blinder decomposition for SRS vs. RST - PTSD outcome*

|  | **Standardized estimate β (95% CI)** | **S.E.** | **p-value** |
| --- | --- | --- | --- |
| *Decomposition* |  |  |  |
| Endowments | -2.251 (-3.943, -0.558) | 0.864 | 0.009 |
| Coefficients | 3.125 (-0.129, 6.379) | 1.660 | 0.060 |
| Interaction | 1.169 (-1.010, 3.349) | 1.112 | 0.293 |
| *Endowments (explained)* |  |  |  |
| Trauma exposure | -2.661 (-4.067, -1.255) | 0.717 | 0.000 |
| Employment: Employed | 0.145 (-0.399, 0.690) | 0.278 | 0.601 |
| Education: High | -0.000 (-0.193, 0.193) | 0.099 | 1.000 |
| Age | 0.015 (-0.335, 0.365) | 0.179 | 0.933 |
| Family status: In relationship | -0.123 (-0.680, 0.435) | 0.284 | 0.666 |
| Gender: Male | 0.373 (-0.449, 1.195) | 0.419 | 0.374 |
| *Coefficients (unexplained)* |  |  |  |
| Trauma exposure | -4.373 (-9.014, 0.268) | 2.368 | 0.065 |
| Employment: Employed | 1.278 (-0.982, 3.539) | 1.153 | 0.268 |
| Education: High | 0.089 (-7.678, 7.855) | 3.962 | 0.982 |
| Age | 3.731 (-8.717, 16.179) | 6.351 | 0.557 |
| Family status: In relationship | -1.614 (-4.401, 1.173) | 1.422 | 0.256 |
| Gender: Male | 0.395 (-3.348, 4.139) | 1.910 | 0.836 |

*Note.* SRS = Syrians residing in Syria, RST = Refugees from Syria resettled in Turkey, PTSD = Posttraumatic stress disorder. CI = Confidence interval, S.E. = Standard error. Significance levels: * *p* ≤ 0.05, ** *p* ≤ 0.01, *** *p* ≤ 0.001.

RST vs. SRS - Oaxaca-Blinder decomposition results - depressive symptoms

Depression severity was higher in the RST group (n = 199, M = 16.45) compared with the SRS group (n = 236, M = 17.02), with a mean difference of Δ ≈ 0.57. Oaxaca–Blinder decomposition indicated that approximately 33% of this difference was explained by group differences in observed characteristics (endowments), 21% by differences in coefficients, and 46% by interaction effects.

**S4**

*Oaxaca-Blinder decomposition for SRS vs. RST - depression outcome*

|  | **Standardized estimate β (95% CI)** | **S.E.** | **p-value** |
| --- | --- | --- | --- |
| *Decomposition* |  |  |  |
| Endowments | -0.535 (-1.146, 0.076) | 0.312 | 0.086 |
| Coefficients | 0.346 (-0.844, 1.535) | 0.607 | 0.569 |
| Interaction | 0.763 (-0.041, 1.568) | 0.411 | 0.063 |
| *Endowments (explained)* |  |  |  |
| Trauma exposure | -0.584 (-1.080, -0.087) | 0.253 | 0.021 |
| Employment: Employed | -0.003 (-0.199, 0.192) | 0.100 | 0.972 |
| Education: High | -0.000 (-0.147, 0.147) | 0.075 | 0.998 |
| Age | 0.034 (-0.111, 0.178) | 0.074 | 0.648 |
| Family status: In relationship | -0.137 (-0.378, 0.103) | 0.123 | 0.263 |
| Gender: Male | 0.156 (-0.156, 0.468) | 0.159 | 0.327 |
| *Coefficients (unexplained)* |  |  |  |
| Trauma exposure | -1.815 (-3.650, 0.020) | 0.936 | 0.052 |
| Employment: Employed | -0.396 (-1.234, 0.443) | 0.428 | 0.355 |
| Education: High | 0.585 (-2.190, 3.359) | 1.416 | 0.679 |
| Age | 3.421 (-1.184, 8.026) | 2.350 | 0.145 |
| Family status: In relationship | -0.861 (-1.859, 0.137) | 0.509 | 0.091 |
| Gender: Male | 0.202 (-1.171, 1.574) | 0.700 | 0.773 |

*Note.* SRS = Syrians residing in Syria, RST = Refugees from Syria resettled in Turkey. CI = Confidence interval, S.E. = Standard error. Significance levels: * *p* ≤ 0.05, ** *p* ≤ 0.01, *** *p* ≤ 0.001.

RSG vs. RST - Oaxaca-Blinder decomposition results - trauma symptoms

PTSD severity was higher in the RSG group (n = 254, M = 44.75) compared with the RST group (n = 199, M = 41.13), with a mean difference of Δ ≈ 3.62. Oaxaca–Blinder decomposition indicated that approximately 31% of this difference was explained by group differences in observed characteristics (endowments), 53% by differences in coefficients, and 16% by interaction effects.

**S5**

*Oaxaca-Blinder decomposition for RSG vs. RST - PTSD outcome*

|  | **Standardized estimate β (95% CI)** | **S.E.** | **p-value** |
| --- | --- | --- | --- |
| *Decomposition* |  |  |  |
| Endowments | -1.636 (-2.934, -0.339) | 0.662 | 0.013 |
| Coefficients | -2.829 (-5.956, 0.299) | 1.596 | 0.076 |
| Interaction | 0.843 (-1.477, 3.163) | 1.184 | 0.476 |
| *Endowments (explained)* |  |  |  |
| Trauma exposure | -0.126 (-0.662, 0.409) | 0.273 | 0.644 |
| Employment: Employed | 0.030 (-0.372, 0.432) | 0.205 | 0.883 |
| Education: High | -0.422 (-0.924, 0.080) | 0.256 | 0.099 |
| Age | -0.703 (-1.464, 0.058) | 0.388 | 0.070 |
| Family status: In relationship | -0.088 (-0.399, 0.224) | 0.159 | 0.581 |
| Gender: Male | -0.328 (-0.930, 0.274) | 0.307 | 0.286 |
| *Coefficients (unexplained)* |  |  |  |
| Trauma exposure | 4.999 (1.035, 8.962) | 2.022 | 0.013 |
| Employment: Employed | -0.445 (-2.060, 1.170) | 0.824 | 0.589 |
| Education: High | 3.156 (-2.598, 8.909) | 2.935 | 0.282 |
| Age | -7.548 (-20.988, 5.892) | 6.857 | 0.271 |
| Family status: In relationship | -0.049 (-3.021, 2.923) | 1.516 | 0.974 |
| Gender: Male | 0.017 (-2.615, 2.649) | 1.343 | 0.990 |

*Note.* RSG = Refugees from Syria resettled in Germany, RST = Refugees from Syria resettled in Turkey, PTSD = Posttraumatic stress disorder. CI = Confidence interval, S.E. = Standard error. Significance levels: * *p* ≤ 0.05, ** *p* ≤ 0.01, *** *p* ≤ 0.001.

RSG vs. SRS - Oaxaca-Blinder decomposition results - depressive symptoms

Depression severity was higher in the RSG group (n = 254, M = 17.08) compared with the RST group (n = 199, M = 16.45), with a mean difference of Δ ≈ 0.63. Oaxaca–Blinder decomposition indicated that approximately 47% of this difference was explained by group differences in observed characteristics (endowments), 42% by differences in coefficients, and 11% by interaction effects.

**S6**

*Oaxaca-Blinder decomposition for RSG vs. RST - depression outcome*

|  | **Standardized estimate β (95% CI)** | **S.E.** | **p-value** |
| --- | --- | --- | --- |
| *Decomposition* |  |  |  |
| Endowments | -0.376 (-0.847, 0.094) | 0.240 | 0.117 |
| Coefficients | -0.341 (-1.621, 0.939) | 0.653 | 0.602 |
| Interaction | 0.086 (-0.793, 0.965) | 0.448 | 0.849 |
| *Endowments (explained)* |  |  |  |
| Trauma exposure | -0.021 (-0.142, 0.100) | 0.062 | 0.733 |
| Employment: Employed | -0.147 (-0.363, 0.070) | 0.111 | 0.185 |
| Education: High | -0.116 (-0.277, 0.045) | 0.082 | 0.159 |
| Age | -0.142 (-0.415, 0.130) | 0.139 | 0.305 |
| Family status: In relationship | 0.043 (-0.093, 0.179) | 0.070 | 0.536 |
| Gender: Male | 0.007 (-0.209, 0.222) | 0.110 | 0.953 |
| *Coefficients (unexplained)* |  |  |  |
| Trauma exposure | 1.304 (-0.413, 3.022) | 0.876 | 0.137 |
| Employment: Employed | 0.439 (-0.182, 1.060) | 0.317 | 0.166 |
| Education: High | -0.580 (-2.867, 1.707) | 1.167 | 0.619 |
| Age | -3.084 (-8.130, 1.961) | 2.574 | 0.231 |
| Family status: In relationship | 0.940 (-0.185, 2.065) | 0.574 | 0.101 |
| Gender: Male | -0.438 (-1.469, 0.593) | 0.526 | 0.405 |

*Note.* RSG = Refugees from Syria resettled in Germany, RST = Refugees from Syria resettled in Turkey. CI = Confidence interval, S.E. = Standard error. Significance levels: * *p* ≤ 0.05, ** *p* ≤ 0.01, *** *p* ≤ 0.001.

RSG vs. SRS - Oaxaca-Blinder decomposition results - trauma exposure

Trauma exposure was higher in the RSG group (n = 254, M = 6.87) compared with the SRS group (n = 236, M = 4.61), with a mean difference of Δ ≈ 2.27. Oaxaca–Blinder decomposition indicated that approximately 5% of this difference was explained by group differences in observed characteristics (endowments), 92% by differences in coefficients, and 3% by interaction effects.

**S7**

*Oaxaca-Blinder decomposition for SRS vs. RSG - trauma exposure*

|  | **Standardized estimate β (95% CI)** | **S.E.** | **p-value** |
| --- | --- | --- | --- |
| *Decomposition* |  |  |  |
| Endowments | -0.112 (-0.540, 0.315) | 0.218 | 0.606 |
| Coefficients | -2.082 (-2.944, -1.219) | 0.440 | 0.000 |
| Interaction | -0.074 (-0.617, 0.469) | 0.277 | 0.789 |
| *Endowments (explained)* |  |  |  |
| Employment: Employed | 0.013 (-0.074, 0.100) | 0.044 | 0.775 |
| Education: High | -0.030 (-0.161, 0.102) | 0.067 | 0.660 |
| Age | -0.397 (-0.720, -0.073) | 0.165 | 0.016 |
| Family status: In relationship | 0.312 (0.061, 0.563) | 0.128 | 0.015 |
| Gender: Male | -0.011 (-0.086, 0.065) | 0.038 | 0.780 |
| *Coefficients (unexplained)* |  |  |  |
| Employment: Employed | 0.340 (-0.137, 0.816) | 0.243 | 0.162 |
| Education: High | -1.197 (-3.133, 0.739) | 0.988 | 0.225 |
| Age | -1.854 (-4.856, 1.148) | 1.532 | 0.226 |
| Family status: In relationship | 0.409 (-0.274, 1.092) | 0.348 | 0.241 |
| Gender: Male | -0.073 (-0.740, 0.594) | 0.340 | 0.830 |

*Note.* SRS = Syrians residing in Syria, RSG = Refugees from Syria resettled in Germany. CI = Confidence interval, S.E. = Standard error. Significance levels: * *p* ≤ 0.05, ** *p* ≤ 0.01, *** *p* ≤ 0.001.

RST vs. SRS - Oaxaca-Blinder decomposition results - trauma symptoms

Trauma exposure was higher in the RST group (n = 199, M = 6.65) compared with the SRS group (n = 236, M = 4.61), with a mean difference of Δ ≈ 2.05. Oaxaca–Blinder decomposition indicated that approximately 13% of this difference was explained by group differences in observed characteristics (endowments), 76% by differences in coefficients, and 11% by interaction effects.

**S8**

*Oaxaca-Blinder decomposition for SRS vs. RST - trauma exposure*

|  | **Standardized estimate β (95% CI)** | **S.E.** | **p-value** |
| --- | --- | --- | --- |
| *Decomposition* |  |  |  |
| Endowments | -0.333 (-0.683, 0.018) | 0.179 | 0.063 |
| Coefficients | -2.007 (-2.876, -1.139) | 0.443 | 0.000 |
| Interaction | 0.292 (-0.110, 0.695) | 0.205 | 0.155 |
| *Endowments (explained)* |  |  |  |
| Employment: Employed | -0.008 (-0.178, 0.162) | 0.087 | 0.923 |
| Education: High | -0.000 (-0.090, 0.090) | 0.046 | 0.999 |
| Age | -0.008 (-0.125, 0.108) | 0.060 | 0.889 |
| Family status: In relationship | -0.018 (-0.177, 0.142) | 0.081 | 0.828 |
| Gender: Male | -0.298 (-0.581, -0.015) | 0.144 | 0.039 |
| *Coefficients (unexplained)* |  |  |  |
| Employment: Employed | 0.175 (-0.519, 0.868) | 0.354 | 0.621 |
| Education: High | -1.002 (-3.642, 1.638) | 1.347 | 0.457 |
| Age | 0.844 (-2.957, 4.645) | 1.939 | 0.663 |
| Family status: In relationship | -0.467 (-1.175, 0.241) | 0.361 | 0.196 |
| Gender: Male | -0.873 (-1.839, 0.092) | 0.492 | 0.076 |

*Note.* SRS = Syrians residing in Syria, RST = Refugees from Syria resettled in Turkey. CI = Confidence interval, S.E. = Standard error. Significance levels: * *p* ≤ 0.05, ** *p* ≤ 0.01, *** *p* ≤ 0.001.

RSG vs. RST - Oaxaca-Blinder decomposition results - trauma exposure

Trauma exposure was higher in the RSG group (n = 254, M = 6.87) compared with the RST group (n = 199, M = 6.65), with a mean difference of Δ ≈ 0.22. Oaxaca–Blinder decomposition indicated that approximately 24% of this difference was explained by group differences in observed characteristics (endowments), 37% by differences in coefficients, and 39% by interaction effects.

**S9**

*Oaxaca-Blinder decomposition for RSG vs. RST - trauma exposure*

|  | **Standardized estimate β (95% CI)** | **S.E.** | **p-value** |
| --- | --- | --- | --- |
| *Decomposition* |  |  |  |
| Endowments | -0.235 (-0.681, 0.211) | 0.227 | 0.301 |
| Coefficients | -0.366 (-1.463, 0.730) | 0.559 | 0.512 |
| Interaction | 0.381 (-0.404, 1.166) | 0.401 | 0.342 |
| *Endowments (explained)* |  |  |  |
| Employment: Employed | -0.064 (-0.206, 0.078) | 0.073 | 0.377 |
| Education: High | -0.029 (-0.167, 0.108) | 0.070 | 0.675 |
| Age | -0.333 (-0.599, -0.067) | 0.136 | 0.014 |
| Family status: In relationship | 0.124 (-0.076, 0.323) | 0.102 | 0.225 |
| Gender: Male | 0.068 (-0.107, 0.243) | 0.089 | 0.445 |
| *Coefficients (unexplained)* |  |  |  |
| Employment: Employed | 0.209 (-0.298, 0.717) | 0.259 | 0.419 |
| Education: High | -0.298 (-2.502, 1.906) | 1.124 | 0.791 |
| Age | -2.796 (-7.259, 1.668) | 2.277 | 0.220 |
| Family status: In relationship | 0.953 (0.057, 1.849) | 0.457 | 0.037 |
| Gender: Male | 0.587 (-0.203, 1.378) | 0.403 | 0.145 |

*Note.* RSG = Refugees from Syria resettled in Germany, RST = Refugees from Syria resettled in Turkey. CI = Confidence interval, S.E. = Standard error. Significance levels: * *p* ≤ 0.05, ** *p* ≤ 0.01, *** *p* ≤ 0.001.

**References**

1. Kim, C. (2010). Decomposing the Change in the Wage Gap Between White and Black Men Over Time, 1980-2005: An Extension of the Blinder-Oaxaca Decomposition Method. *Sociological Methods & Research*, *38*(4), 619–651. <https://doi.org/10.1177/0049124110366235>
2. Jann, B. (2008). The blinder–oaxaca decomposition for linear regression models. *The Stata Journal: Promoting Communications on Statistics and Stata*, *8*(4), 453–479. https://doi.org/10.1177/1536867X0800800401
3. Hlavac, M. (2014). oaxaca: Blinder-Oaxaca Decomposition in R. *SSRN Electronic Journal*. <https://doi.org/10.2139/ssrn.2528391>
